# Ingested Chicken Bone (Xiphoid Process) in the Anal Canal: A Case Report and Literature Review

**DOI:** 10.7759/cureus.35060

**Published:** 2023-02-16

**Authors:** Ahmed F Alkandari, Husain M Alsarraf, Mohammed F Alkandari

**Affiliations:** 1 Department of Anatomy, Kuwait University, Jabriya, KWT; 2 Department of Surgery, Al-Adan Hospital, Al-Ahmadi, KWT; 3 Medicine, University College Dublin, Dublin, IRL; 4 Department of Dentistry, Sabah Al-Ahmad Clinic, Sabah Al-Ahmad, KWT

**Keywords:** swallowing, ingestion, foreign body, xiphoid process, chicken bone

## Abstract

Accidental foreign body (FB) ingestion is common in the elderly, particularly edentulous and denture wearers. The most commonly ingested FBs are food-related, including fish and chicken bones. While small FBs can pass through the gastrointestinal tract without any complications, large or irregular-shaped FBs usually cause complications. These complications include choking, ulceration, perforation, fistula, abscess formation, or even death. Ingestion of a large chicken bone that reaches the anal canal without causing injury is extremely rare.

We present a rare case of accidental chicken bone (xiphoid process) ingestion that manifested itself by projecting from the anus while defecating. Interestingly, the patient did not experience any food choking or abdominal pain, nor did she have any type of dementia. However, the patient could not grind food properly due to the loss of her upper molar teeth, which made her develop a habit of rapid swallowing. On examination, the chicken bone was seen within the anus with a sharp edge embedded in the mucosal wall. While a plain pelvic X-ray failed to display the FB, a pelvic CT scan with a 3D illustration showed its dimensions (5.0 x 2.5 x 3 cm). A plain pelvic CT scan confirmed the presence of the bone-contrast FB within the anal canal without injuring the surrounding anatomical structures. The FB was gently manipulated and successfully extracted after administering 5 ml of lidocaine gel enema. The patient was counseled regarding proper chewing habits, and she was referred to a dental clinic for a professional assessment. Seven days later, the patient was seen again at the clinic for a follow-up and was found to have an uneventful recovery. Maintaining good dental health and proper eating habits, as well as increasing awareness among edentulous individuals and denture wearers, are required to prevent accidental FB ingestions.

## Introduction

Induced ingestion of foreign body (FB) materials is common among prisoners [1], psychotic patients [2], and females with pica, particularly during pregnancy [3]. However, accidental FB ingestion occurs due to several reasons and risk factors. Accidental FB ingestion is mostly seen in children due to their normal suckling behavior, whereas in adults, it occurs mostly in alcoholics and edentulous individuals [4,5]. Among the FBs ingested in adults, dentures [6] and toothpicks [7], together with fish and chicken bones [7-9], are the most common.

Generally, small FBs (i.e., fragments of or long-structured chicken bones) can pass to the stomach, proceed through the intestines, and be expelled from the gastrointestinal (GI) tract without any consequences [5]. However, in rare cases, ingested small chicken bones may reach the rectum or anal canal, where rectal perforation and infection can occur. In contrast, large FBs (i.e., intact or irregular-shaped chicken bones) are less likely to pass the esophagus [10]. If they pass through the esophageal constrictions, the ingested FBs are usually found lodged in the anatomical strictures (i.e., ileocecal valve, sigmoido-rectal junction, etc.) or pathological pouches (i.e., hernia, diverticulum, etc.), leading to obstruction, perforation, peritonitis, or even death if neglected. A literature review regarding the consequences of chicken bone ingestion is provided in the discussion section below.

To the best of our knowledge, this is the first case report to describe the ingestion of an intact xiphoid bone of a chicken that reached the anal canal without causing any complications. We present a case of chicken bone ingestion (intact xiphoid process), measuring 5.0 x 2.5 x 3.0 cm in size, which was successfully extracted from the anal canal without further complications.

## Case presentation

A 51-year-old female presented to our department complaining of a hard, sharp object projecting from her anus while defecating. The patient denied any history of food choking or abdominal pain, and her family confirmed that she does not have dementia. However, she had a history of chronic constipation (three times/week; hard feces), internal piles, and an anal fissure. Upon questioning about dietary habits, the patient reported reduced mastication and rapid swallowing due to the loss of upper molar teeth. Also, she had recently placed an upper dental bridge over the incisors for cosmetic reasons.

**Figure 1:**
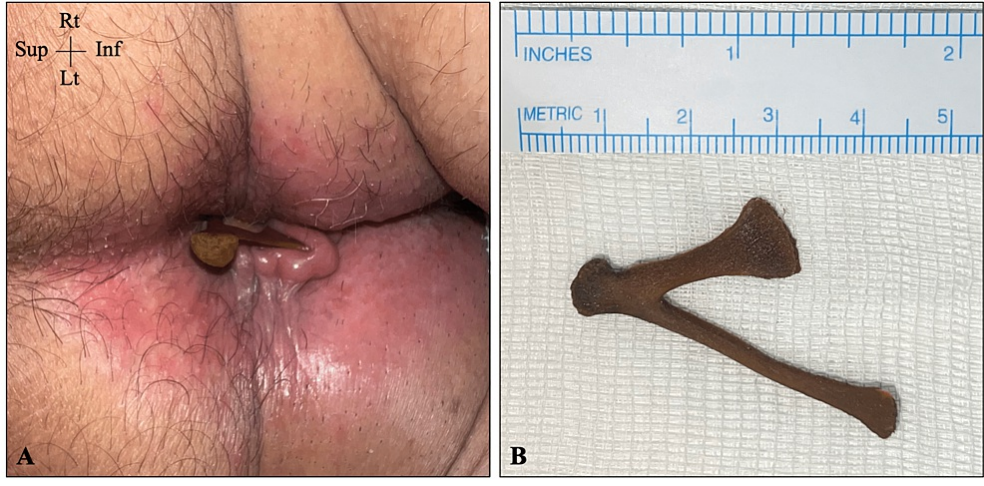
The ingested chicken bone (A) The ingested FB protruding from the anus (note the swollen anal mucosa due to an embedding bony edge). (B) The ingested FB with its measurement after being extracted from the patient FB: foreign body; Sup: superior; Inf: inferior; Rt: right; Lt: left

**Figure 2:**
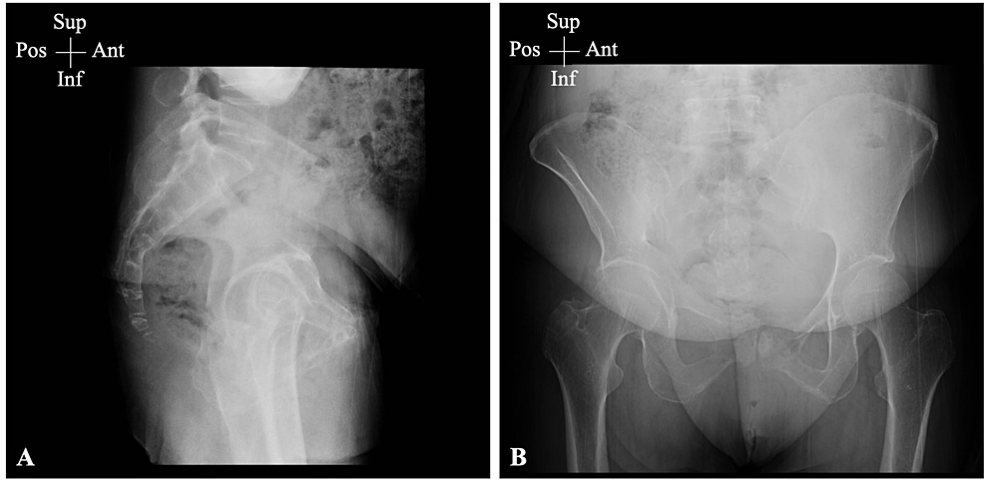
Plain pelvic X-ray Lateral (A) and posteroanterior (B) views of the pelvic plain X-ray. Although the ingested FB was of bone contrast, a plain X-ray failed to provide its shape and dimensions FB: foreign body; Sup: superior; Inf: inferior; Ant: anterior; Pos: posterior

**Figure 3:**
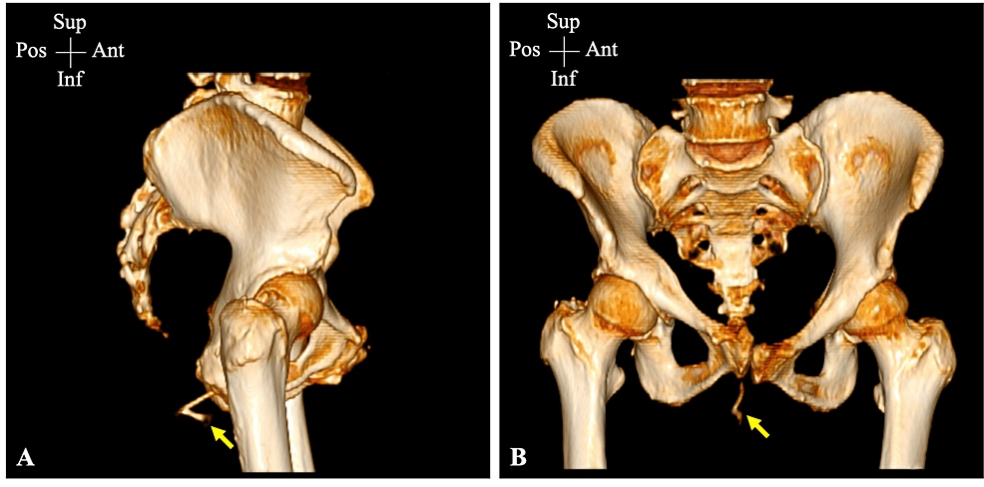
Pelvic CT scan with 3D illustration Lateral (A) and posteroanterior (B) views of pelvic CT scan with a 3D illustration. The ingested FB was clearly demonstrated (yellow arrow), having a V-shaped structure with a bone contrast without sharp components. The thinner long and wider short limbs measure 5.0 and 2.5 cm, respectively CT: computed tomography; FB: foreign body; Sup: superior; Inf: inferior; Ant: anterior; Pos: posterior

**Figure 4:**
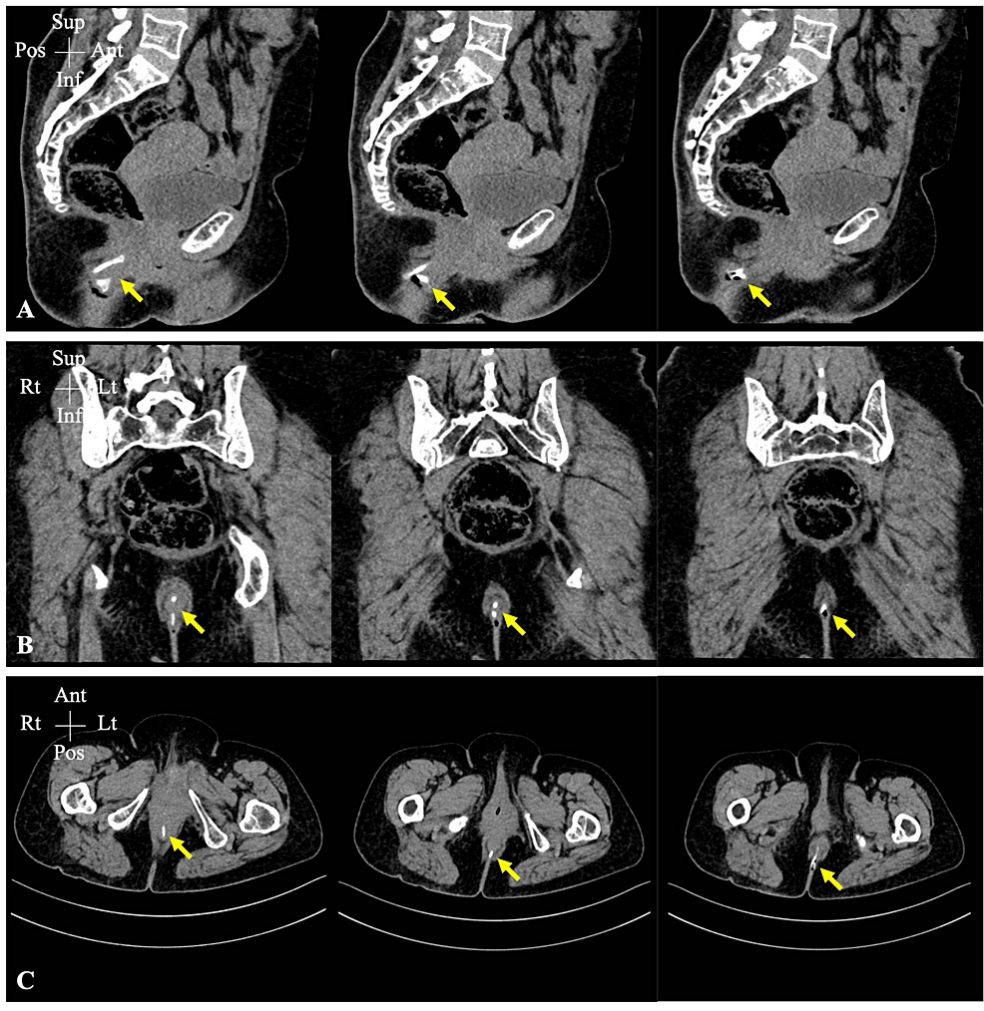
Plain pelvic CT scan Lateral (A), posteroanterior (B), and cross-sectional (C) views of plain pelvic CT scan. The ingested FB was demonstrated (yellow arrow), with the short limb projecting outside the anus, while the long limb was within the anal canal. There were no definite sharp components and no injury to the surrounding structures CT: computed tomography; FB: foreign body; Sup: superior; Inf: inferior; Ant: anterior; Pos: posterior; Rt: right; Lt: left

On examination, a triangular-shaped bony structure was found projecting from the anus with a sharp edge embedded in the mucosal wall (Figure 1A). A plain pelvic X-ray failed to display the FB or its dimensions (Figure 2). Therefore, a pelvic CT scan with a 3D illustration was performed, which showed a V-shaped FB that had thinner long and wider short limbs measuring 5.0 and 2.5 cm, respectively (Figure 3). A plain pelvic CT scan showed that the short limb was projecting outside the anus, while the long limb was seen within the anal canal with no definite sharp components and no injury to the surrounding structures (Figure 4).

After reassuring the patient, 5 ml of lidocaine gel enema was slowly administered to numb the area, expand the mucosal wall, and allow for the easy removal of the FB. The FB was then gently manipulated and extracted. Thereafter, a digital rectal examination showed no signs of FB-induced injury. The patient was advised about proper chewing habits, and she was referred to a dental clinic for a professional assessment. After seven days, the patient was followed up at the clinic and her recovery was uneventful.

## Discussion

The xiphoid bone of chicken

The ingested bone in our patient was from an avian skeleton of a chicken, specifically a xiphoid process with its both lateral processes (Figure 1B). It had a characteristic V shape with a maximum width of 3.0 cm and foot-like expansions on each limb. The wider limb was 2.5 cm in length and had a large pedal expansion (the upper oblique xiphoid process), while the thinner one measured 4.0 cm with a smaller pedal expansion (the lower long xiphoid process).

**Figure 5:**
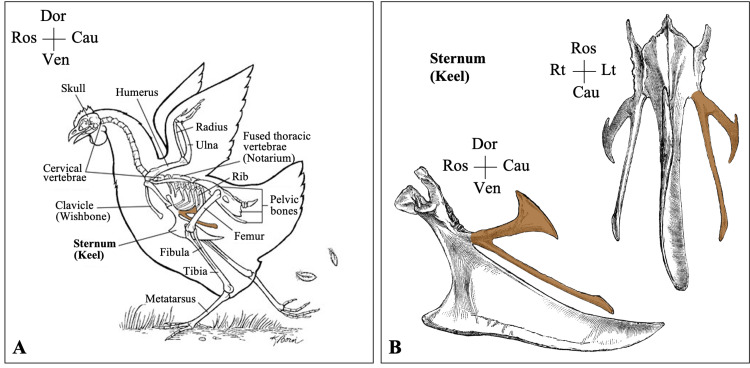
The xiphoid bone of chicken (A) The skeleton of chicken (copyright © John Wiley & Sons, Inc; illustrated by Kathryn Born, used with permission) [13]. (B) The sternum of chicken (copyright © ClipArt ETC, used with permission) [14]. The xiphoid bone is shadowed in brown Dor: dorsal; Ven: ventral; Ros: rostral; Cau: caudal; Rt: right; Lt, left

In chickens, the sternum is made up of cartilage at birth and develops into bone throughout adulthood. It begins to ossify from five centers, one for the keel located in the center, and two pairs for the lateral parts of the sternum located anteriorly and posteriorly. The two antero-lateral centers (precessus lateralis anterior) form the pleurostron, which is equivalent to the manubrium or manubristernii in humans. The central median center forms the lophosteon, which is equivalent to the body of the sternum in humans. The two postero-lateral centers (precessus lateralis posterior) form the metosteon, which is equivalent to the xiphoid bone or xiphisternum in humans. As opposed to human anatomy, the xiphoid process in mammals has many divisions. The xiphoid bone in chickens is divided into two lateral parts (Figure 5). Each part is further divided into two lateral processes that end in a foot-like expansion. The upper oblique xiphoid process terminates in a broad end just behind the last sternal rib, while the lower long xiphoid processes run downward and backward [11,12].

Risk factors of FB ingestion

The role of dentistry in accidental FB ingestion has been established [15]. Wearing dentures is a common risk factor associated with FB ingestion in adults. Ganesh et al. (2021) have reported 34 cases of swallowed dental prostheses [6]. This may be due to the fact that dentures themselves are commonly ingested FBs [6] or because they cover the sensitive palate allowing any FB to be ingested without being felt [16,17]. In addition, the role of the teeth in preventing the ingestion of large food particles or FBs is well established. The periodontal mechanoreceptors allow for encoding the tooth load when food comes into contact with the teeth [18]. In turn, the molar teeth contribute to the grinding process, which breaks down large food particles into smaller portions and mixes them with saliva to soften them. Edentulous people are at increased risk of FB ingestion because the relatively increased volume of their oropharynx allows large food portions or FBs to be contained in their mouths. Also, loss of teeth sensation and grinding allows FB materials to be propelled to the esophagus without being held in place. Bad food-eating habits, such as over-eating and rapid swallowing, can predispose to accidental FB ingestion. These habits are often acquired from teeth decays, which leads to immobilization reflux to avoid biting over the location of the pain. Another factor that contributes to FB ingestion and passage through the esophagus is the habit of rapid swallowing. While peristalsis is induced by the contractions of both circular and longitudinal smooth muscles of the GI tract, the circular muscle contraction causes axial shortening of the esophagus due to its spiral orientation [19]. On the other hand, rapid swallowing temporarily suppresses the peristalsis, which, in turn, leads to maximal dilatation of the esophagus [20]. Since swallowing initiates relaxation, a greater relaxation causes a greater luminal distention of the esophagus [19], which explains the passage of large FB through the esophagus without causing damage.

Consequences of chicken bone ingestion

Ingestion of chicken bone can result in serious complications. Death was reported in five cases, in which the ingested chicken bone perforated the esophagus and injured the major adjacent vessels [21-25]. Most of the reported cases regarding chicken bone ingestion involve a fragmented piece or linear-shaped FB. Ingestion of configured-shaped FBs (i.e., V-, Y- or L-shaped FBs) has only been reported in a few cases. Ingestion of xiphoid bone has only been reported twice in the literature [21,26]. The first report was by Russo et al. (1986), in which an 81-year-old edentulous woman collapsed while eating chicken. Autopsy findings showed a perforated esophagus and a lacerated left common carotid artery. The chicken bone was consistent with the xiphoid bone of the chicken, although it was not an intact piece of bone [21]. The second report was by Chen et al. (2016), in which an 84-year-old woman was found to have an impacted chicken bone in the sigmoid colon while performing a colonoscopy for her episodic abdominal pain. The chicken bone, which was consistent with the xiphoid bone of the chicken, was broken into two pieces with the aid of Nd:YAG laser and then extracted with rat-tooth-grasping forceps [26]. Ingestion of the wishbone (clavicle) of chicken has been reported three times in the literature [25,27,28]. The ingested wishbone reported by Hoxha et al. (2009) resulted in ileal perforation. Although the wishbone was partially broken, its shape and dimension were similar to the xiphoid bone ingested in our patient [27].

Several complications result from chicken bone ingestion based on the size and shape of the ingested bone. Large chicken bones can lead to luminal obstruction, especially at the anatomical strictures. On the other hand, linear chicken bones can pass the anatomical strictures but are prone to enter the pathological pouches, such as the colonic diverticula, Meckel’s diverticulum, and hernial sac. Ingested linear chicken bones usually become impacted across two diverticula or one diverticulum and the luminal wall in the case of colonic diverticulosis, leading to diverticulitis and perforation [29]. Similarly, complications of chicken bone ingestion are frequently evident in Meckel’s diverticulum [30]. Moreover, ingested chicken bones commonly become impacted in the intestinal loop within a hernial sac [31], leading to strangulation and perforation. Fragmented or sharp-edged chicken bones can cause luminal perforation anywhere in the GI tract [32], including the appendix [33]. In addition, ingested sharp chicken bones can result in fistula formation between the lumen of the GI tract and surrounding viscera, even if they were expelled within the feces [34].

Esophageal perforation due to ingested chicken bone has been frequently reported to cause aorto-esophageal fistula because of the close anatomical relation [35]. Similarly, subclavian-esophageal fistula secondary to ingested chicken bone is also reported in the literature [36]. In the sigmoid colon, diverticulosis is a risk factor for perforation after chicken bone ingestion. Sigmoido-vesical fistula is a common presentation of chicken bone ingestion at the sigmoid colon [37]. Caes et al. (1988) reported a case of an aorto-colic fistula between the descending aorta and sigmoid colon that resulted in rectal bleeding and required surgical intervention [38]. Cash et al. (2004) reported a case of chicken bone ingestion that resulted in an anorectal fistula and subsequent ischiorectal abscess [39]. The chicken bone-induced fistula formation may present with abscess or massive bleeding. In the case of sealed perforation, migration of chicken bone to the surrounding structures can occur with or without FB reaction or abscess formation. Afghani et al. (2016) reported a case of an ingested chicken bone found in the bronchial tree, which was surgically treated [40]. Demirhan et al. (2016) reported a case of ingested chicken bone migrated to the subcutaneous tissue of the neck [41]. Elwerfelli et al. (2020) reported a case of asymptomatic chicken bone ingestion that presented in the pleural cavity and required thoracoscopic removal [42]. Berk and Reit (1971) reported two cases of chicken bone located in the peritoneal cavity and caused intra-abdominal abscesses [43]. Hoff et al. (2021) reported a case of liver abscess due to ingested chicken bone [44].

A few cases have been reported of chicken bone ingestion reaching the rectum and anal canal. Moreira et al. (1975) reported a case of chicken bone ingestion in a denture-wearer patient, which resulted in rectal perforation and necrotizing fasciitis that required extensive surgical debridement [45]. Rectal perforation also occurred in two other patients, in which ischiorectal abscesses necessitated incision and drainage [39,46]. Successful removal of ingested chicken bone in the rectum has only been reported in two cases, in which it was achieved digitally [47] or by clamp [48]. However, failure or inability to remove ingested chicken bone in the rectum can result in recto-sigmoidal resection [49]. Only two cases of ingested chicken bone in the anal canal have been previously reported in the literature [50,51], as shown in Table 1.

**Table 1:** Reported cases of ingested chicken bone in the anal canal

Reference (author, year)	Patient (age, gender)	Chicken bone (length, shape)	Consequences	Management	Predisposing factors
Aduful, 2006 [50]	20, F	4.0 cm, trapezoid	Perforation, fistula, abscess	Fistulotomy	Nil
Choi, 2008 [51]	64, M	2.5 cm, rectangular	Pain	Removed by forceps	Alcohol
Alkandari et al., 2023 (present case)	51, F	5.0 cm, V-shaped (xiphoid bone)	Discomfort	Digitally removed	Dentures

Case discussion

**Figure 6:**
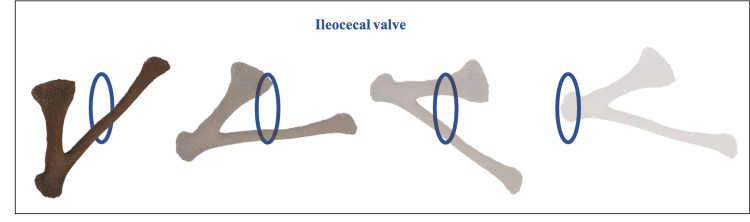
The passage of the ingested xiphoid bone of chicken through the ileocecal valve From right to left is the suggested orientation of the ingested FB to pass through the ileocecal valve without causing an obstruction

There are scarce explanations as to how an intact irregular chicken bone (i.e., xiphoid) can travel all the way from the GI tract and reach the anal verge without causing an injury. Also, it is surprising how a large FB (measuring 5 x 3 x 2.5 cm) can pass the GI tract without causing choking or abdominal pain. In our patient, the loss of upper molar teeth relatively increased the oropharynx and allowed the FB to be contained in the mouth without being felt. Moreover, teeth loss was associated with the inability to grind food properly. This forced the patient to acquire bad food-eating habits like rapid swallowing, which, in turn, facilitated FB swallowing. The esophagus has three constriction points where an ingested FB can stick. The cervical constrictor (also called the upper esophageal sphincter) is caused by the cricopharyngeus muscle, the broncho-aortic constrictor is caused by the left main bronchus and arch of the aorta, and the diaphragmatic constrictor (also called the lower esophageal sphincter) that is caused by the diaphragm [52]. Its normal diameter ranges from 2.0 to 3.3 cm and can reach up to 4.9 cm when maximally dilated [20]. The habit of rapid swallowing allowed the esophagus to maximally dilate, thereby allowing the passage of FB through the esophagus. The cardiac sphincter might be relaxed due to age-related muscle weakness. In the stomach, gastric enzymes may partially digest and soften the bone, making it semi-flexible and lowering the risk of impaction. The FB may have exited the pyloric sphincter due to the presence of food in the stomach, which pushed it from behind, similarly in the small intestine. Although anatomical variations exist, the diameter of the small intestine averages 2.5 cm, and that of the large intestine averages 4.8 cm [53]. The dedendum is the widest part of the small intestine, while the jejunum is thicker and the ileum is the thinnest. Apart from being the thinnest part, the ileum is also the least vascular part of the small intestine, which perhaps makes it the most common location of perforation. The mechanism that often prevents perforation in the intestine seems to be the axial flow of the FB in the lumen, combined with reflex relaxation of the muscle wall, which tends to turn sharp objects around, making the sharp end trail, rather than lead [2]. The ileocecal valve, however, is a tight junction. The terminal ileum is a high-pressure zone since it represents the collection of peristaltic pressure, at which most ingested FB-induced intestinal perforations occur. The orientation of the ingested xiphoid bone plays an essential role in determining the direction of the FB itself, thus allowing or preventing intestinal obstruction. For instance, intestinal obstruction is less likely to occur if the xiphoid bone is directed as illustrated in Figure 6. This is because the acute angle of the bone will gradually widen the valve while passing through it.

In the middle of the transverse colon, ingested FBs become covered by feces [2], thereby facilitating the passage of the ingested objects through the remaining portions of the large bowel and anal canal. Additionally, constipation may allow FB to be accompanied by hard feces while passing through the large bowel, thus concealing its pointed edges. Chronic constipation may also contribute to a slight gradual dilatation of the colon.

Once FB ingestion is suspected, detailed history taking and examination are important. In our case, it was surprising that the patient denied any history of post-prandial choking or abdominal pain, although the family assured us that she does not have dementia. Also, diagnostic imaging is important in confirming the diagnosis of FB ingestion and detecting further complications. Douglas and Sistrom (1991) have reported a case of an ingested chicken bone that perforated the esophagus, which was diagnosed by a CT scan while both X-ray and endoscopy were unremarkable [54]. Similarly, Mesallam (2011) reported a case of an ingested chicken bone embedded in the pyriform fossa of the laryngopharynx, which was poorly visualized by X-ray [55]. In our study, the pelvic CT scan with a 3D illustration was a useful tool to demonstrate the dimensions of the swallowed FB and to exclude any concealed sharp border, which may damage the anal canal during the extraction. CT scan also confirmed no intra-abdominal or -pelvic injury secondary to the ingested FB. The application of lidocaine gel enema in our patient helped to ease the discomfort and facilitate pulling out the object gently.

## Conclusions

It is rare for an intact xiphoid bone of a chicken to be swallowed and travel through the GI tract to reach the anal canal without causing severe injury. This report discusses the possible contributing factors for FB ingestion, passage, and impaction at the anal verge. Also, the management of this rare condition is described. To prevent accidental FB ingestions, good dental health and proper eating habits are needed. Also, raising awareness among edentulous individuals and denture wearers is warranted to avoid such accidents.
